# Occupational eye dose in interventional cardiology procedures

**DOI:** 10.1038/s41598-017-00556-3

**Published:** 2017-04-03

**Authors:** Yoshihiro Haga, Koichi Chida, Yuji Kaga, Masahiro Sota, Taiichiro Meguro, Masayuki Zuguchi

**Affiliations:** 1 Department of Radiological Technology, Faculty of Health Sciences, Tohoku University Graduate School of Medicine, 2-1 Seiryo, Aoba, Sendai 980-8575 Japan; 2 Department of Radiology, Sendai Kousei Hospital, 4-5 Hirose-machi, Aoba-ku, Sendai 980-0873 Japan; 3 Department of Radiation Disaster Medicine, International Research Institute of Disaster Science, Tohoku University, Aramaki Aza-Aoba 468-1, Aoba-ku, Sendai 980-0845 Japan; 4 Department of Cardiovascular Medicine, Sendai Kousei Hospital, 4-5 Hirose-machi, Aoba-ku, Sendai 980-0873 Japan

## Abstract

It is important to measure the radiation dose [3-mm dose equivalent, Hp(3)] in the eye. This study was to determine the current occupational radiation eye dose of staff conducting interventional cardiology procedures, using a novel direct eye dosimeter. We measured the occupational eye dose [Hp(3)] in physicians and nurses in a catheterization laboratory for 6-months. The eye doses [Hp(3)] of 12 physicians (9 with Pb glasses, 3 without), and 11 nurses were recorded using a novel direct eye dosimeter, the DOSIRIS^TM^. We placed dosimeters above and under the glasses. We also estimated the eye dose [0.07-mm dose equivalent] using a neck personal dosimeter. The eye doses among interventional staff ranked in the following order: physicians without Pb glasses > physicians with Pb glasses > nurses. The shielding effect of the glasses (0.07-mm Pb) in a clinical setting was approximately 60%. In physicians who do not wear Pb glasses, the eye dose may exceed the new regulatory limit for IR staff. We found good correlations between the neck dosimeter dose and eye dosimeter dose (inside or outside glasses, R^2^ = 0.93 and R^2^ = 0.86, respectively) in physicians. We recommend that interventional physicians use an eye dosimeter for correct evaluation of the lens dose.

## Introduction

During interventional radiology (IR)/interventional cardiology (IC) procedures, patients and physicians can be injured due to prolonged exposure to X-ray radiation^1–7^. The new recommendation of the International Commission on Radiological Protection (ICRP) for the occupational eye dose limit is an equivalent dose limit in the eye lens of 20 mSv/year, averaged over defined 5-year periods, with no single year exceeding 50 mSv^8, 9^. This is markedly reduced from the previous limit of 150 mSv/year. Therefore, it is essential to evaluate the occupational eye dose and eye protection.

Occupational exposure for IR/IC staff is a critical issue for medical workers^10–17^. However, the exact occupational eye dose in IR/IC staff is not clear. The purpose of this study was to determine the current occupational radiation eye dose [3-mm dose equivalent, Hp(3)] of IC staff conducting interventional cardiology procedures, using a novel direct eye dosimeter. We used dosimeters to experimentally assess whether lead glasses adequately protected the eyes of physicians performing IC procedures.

Furthermore we compared the eye dose measured using the direct eye dosimeter with the evaluated eye dose using a neck personal dosimeter.

## Materials and Methods

### Subject

We evaluated the new eye dosimeter over a 6-month period (September 2015 to February 2016). Doses were monitored over 1-month intervals within this period. We calculated the cumulative 6-month eye dose (the half-year occupational dose) for IC staff (Table 1).

**Table 1:** Summary of our 6-month study.

Staff	*n*	Eye dosimeter dose, Hp(3), (mSv)	Neck badge dose, Hp(0.07), (mSv)	Number of procedures	Total fluoroscopy time (min.)
**Physicians**					
With Pb glasses (*Range*)	*9*	3.1 ± 1.3 (*4.6*–*1.4*)	11.4 ± 6.4 (*18.8*–*1.9*)	134.1 ± 80.2 (*230*–*22*)	1323 ± 771 (*1950*–*164*)
**Physicians**					
Without Pb glasses (*Range*)	*3*	6.3 ± 5.1 (*10.4*–*0.6*)	5.0 ± 2.6 (*7.8*–*2.5*)	84.0 ± 64.2 (*124*–*10*)	1014 ± 606 (*1565*–*364*)
**Nurses**					
(*Range*)	*11*	1.6 ± 1.0 (*3.7*–*0.5*)	2.0 ± 1.2 (*4.1*–*0.4*)	192.7 ± 84.4 (*371*–*104*)	2383 ± 1053 (*3936*–*750*)

mean ± SD.

In Sendai-Kosei Hospital, the occupational radiation exposure (eye dose) of 12 IC physicians and 11 IC nurses during cardiac catheterization and IC procedure was evaluated from September 2015 to February 2016, during which 1707 coronary angiograms and 902 IC procedures such as percutaneous coronary intervention (PCI) were performed at the hospital. During these procedures, the IC staff wear protective aprons (usually 0.35-mm lead (Pb) equivalent).

Nine physicians also wear Pb glasses (which protect against lateral radiation; 0.07 mm Pb, Panorama Shield, Toray, Japan), while three do not. The Pb glasses are lightweight (only 42 g) and comfortable.

We used digital cine angiography X-ray systems with flat-panel detector (Infinix Celeve-i, Toshiba) for all procedures. The number of procedures and the total fluoroscopy time were recorded.

During all procedures, physicians were usually positioned close to the right sides of the patients. In contrast, the distance between nurses and patients was about 3 meter, although some variation occurred.

This study was approved by the ethics committee of our institution (Sendai Kousei Hospital). Informed consent was obtained from all subjects. All PCIs were performed in accordance with the guidelines promulgated by the Japanese Circulation Society^18^.

### Dosimetry

The staff participating in this study used a new dedicated direct eye lens dosimeter (eye dosimeter), the DOSIRIS^™^ (IRSN, France), which specifically measures the eye lens dose [Hp(3)]^12^. The eye dosimeter consists of a thermoluminescent dosimeter sensor (^7^LiF:Mg, Ti), plastic capsule, and adjustable headset. The laboratory at IRSN supplied and calibrated the eye dosimeters. Following each 1-month measurement period, the eye dosimeters were returned to IRSN in France to be read. Dose calibration was performed in IRSN by reference to the national standard.

**Figure 1:**
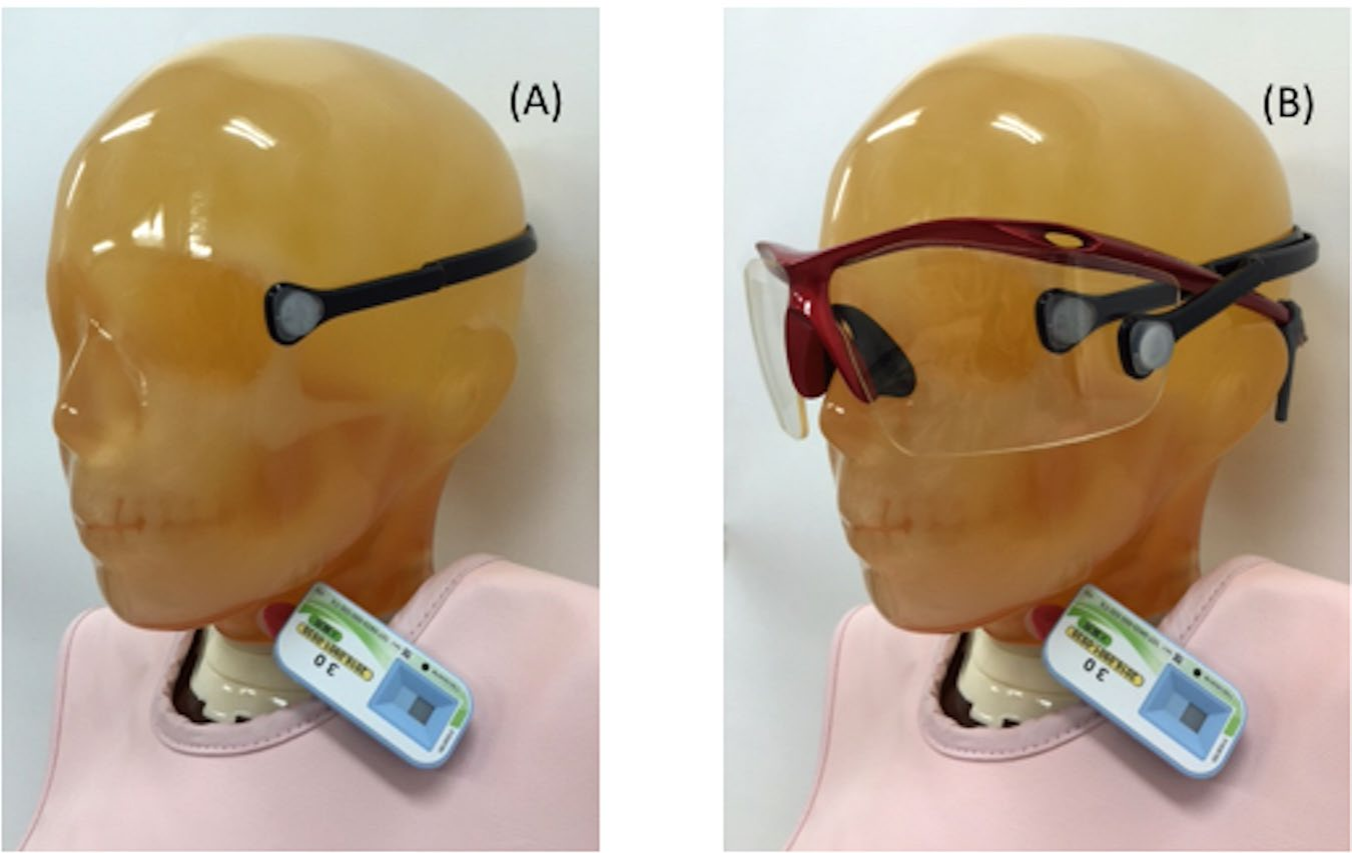
The positions of the dosimeters used during the procedures. The eye dosimeter (DOSIRIS) was worn just lateral to the left eye, and the personal dosimeter (badge) was worn outside the Pb apron to the left of the neck (**A**). In the nine physicians using Pb glasses, an additional eye dosimeter (DOSIRIS) was worn outside the Pb glasses close to the left eye (**B**).

All IC staff wore an eye dosimeter just lateral to the left eye. The nine IC physicians who used Pb glasses wore an additional eye dosimeter outside the Pb glasses close to their left eye (Fig. 1). We also estimated the eye dose in all IC staff using a neck personal dosimeter (Fig. 1). The commercial neck personal dosimeter used was a silver-activated phosphate glass dosimeter [0.07-mm dose equivalent, Hp(0.07), Glass Badge, Chiyoda-Technol, Japan), which was worn outside the personal Pb apron to the left of the neck. The glass badges were returned monthly to Chiyoda Technology for evaluation.

We evaluated the protective effects of Pb glasses in nine physicians by placing eye dosimeters inside and outside the glasses (three physicians did not wear Pb glasses). We also measured the correlation between the eye dose [Hp(3)] and the neck dose [Hp(0.07)] to explore whether it was feasible to estimate the eye dose using a neck dosimeter.

We also determined the estimated annual eye dose (EAED) as follows:

$${EAED}({\rm{mSv}}/{\rm{year}})={dose}\,{measured}\,{over}\,6\,{months}\times 2$$

### Statistics

The Steel–Dwass test was used to compare data (estimated average annual eye dose) among the three groups (physicians who wore neck badges, and those who wore eye dosimeters and also Pb glasses or not). The Wilcoxon signed-rank test was used to compare data (estimated average annual eye dose) between two groups of nurses (those who wore neck and eye dosimeters). Linear regression was employed to evaluate correlations between doses to the neck badges and the eye dosimeters (inside and outside the Pb glasses). Also, correlations between doses to the internal and external dosimeters were sought by linear regression. Finally, correlations between doses to the neck badges and eye dosimeters of nurses were also explored by linear regression. Statistical significance was defined as *p* < 0.05.

## Results

Table 1 summarizes the results of our 6-month study. The eye doses were markedly higher in the physician than in the nurses.

### Physician eye dose

**Figure 2:**
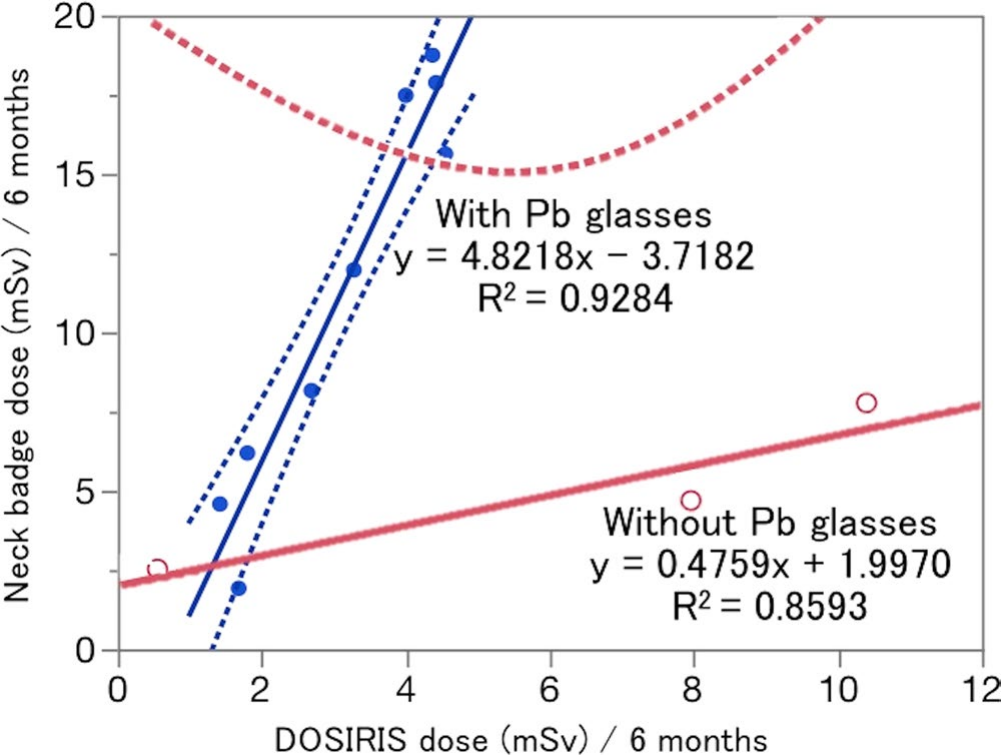
Relationship between the eye dose (DOSIRIS) measurements and neck badge dose measurements for 6 months in 12 IC physicians: nine physicians used Pb glasses and three did not. Dashed line (---): 95% confidence interval.

**Figure 3:**
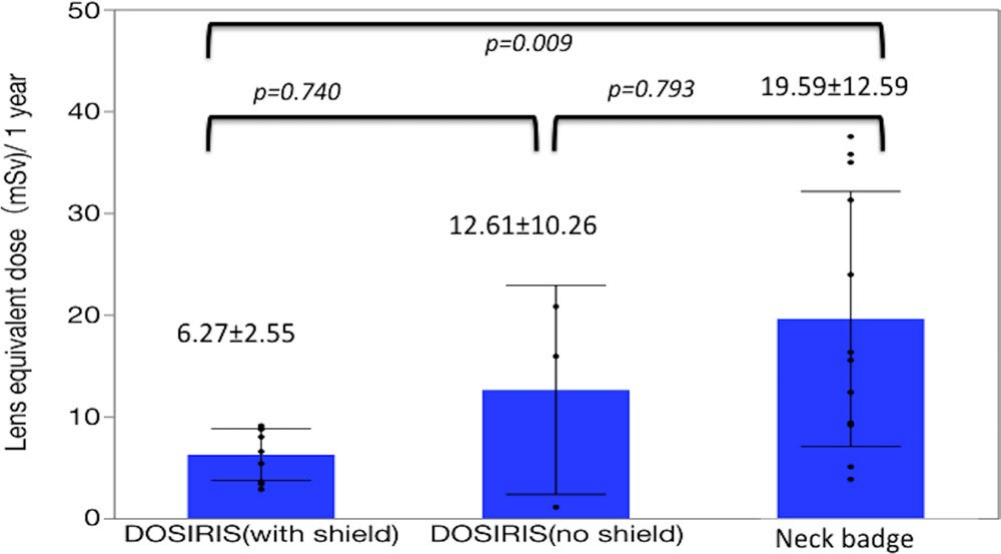
Estimated mean ± SD annual lens dose (EAED) in 12 IC physicians estimated by eye dose (DOSIRIS) measurements (9 physicians used Pb glasses and 3 did not) and neck badge dose measurements.

There were significant correlations between the neck badge dose and the eye dosimeter measurements both with and without Pb glasses (Fig. 2). Figure 3 shows the EAEDs. One physician who did not wear Pb glasses exceeded the equivalent dose limit for the lens (20 mSv/year). The eye dose evaluated using a neck dosimeter tended to be overestimated.

**Figure 4:**
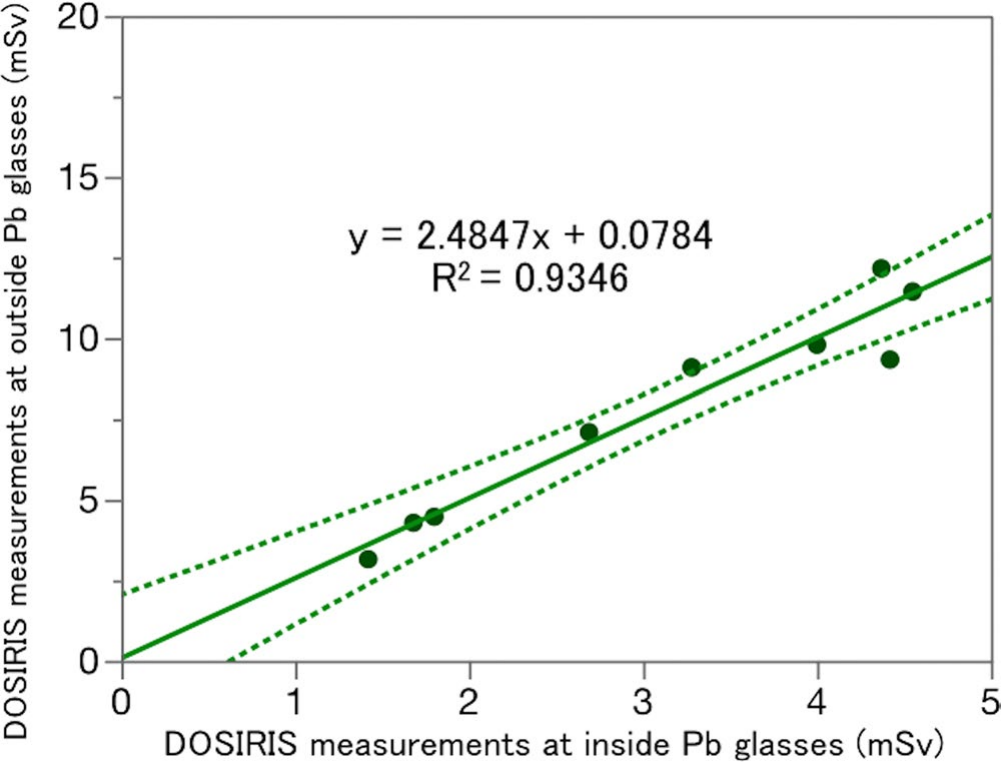
Relationship between the eye dose (DOSIRIS) measurements inside and outside the Pb glasses over 6 months in nine physicians. Dashed line (---): 95% confidence interval.

Figure 4 shows the relationship between the eye dose using eye dosimeter inside the Pb glasses and that using an additional eye dosimeter outside the Pb glasses in the nine IC physicians who used Pb glasses. The correlation between the inside and outside doses was excellent. The mean ± SD radiation doses for inside and outside the Pb glasses were 3.1 ± 1.3 and 7.9 ± 3.3 mSv/6 months, respectively. Therefore, the shielding effect of the Pb glasses was approximately 60% in a clinical IC setting.

### Nurse eye doses

**Figure 5:**
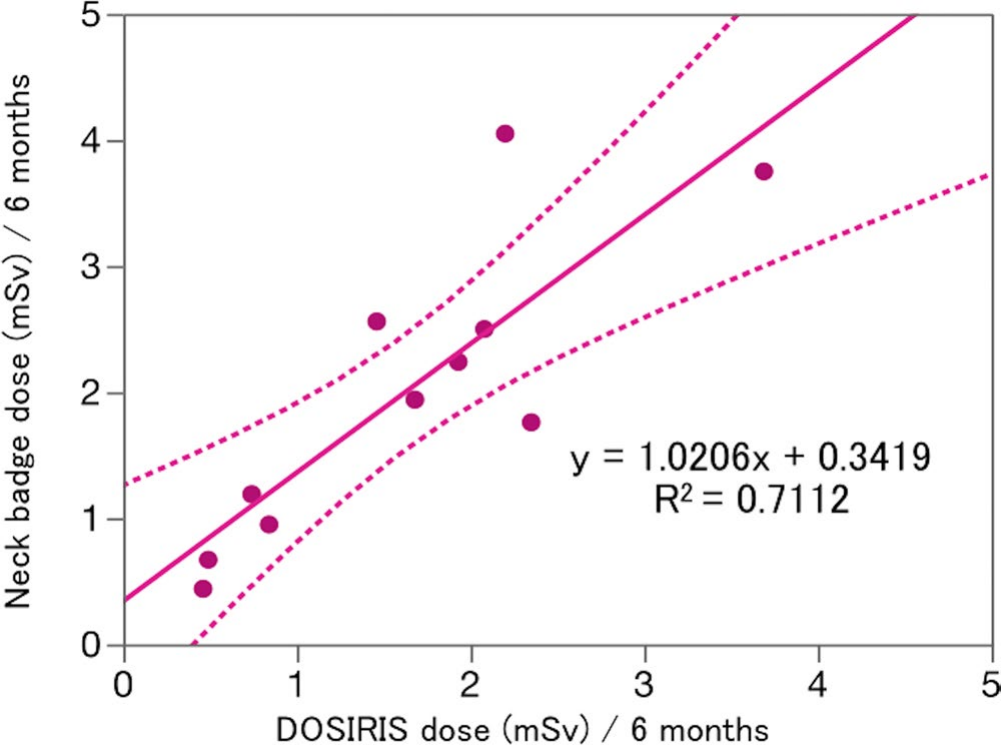
Relationship between the eye dose (DOSIRIS) and neck badge dose measurements over 6 months in 11 IC nurses. Dashed line (---): 95% confidence interval.

**Figure 6:**
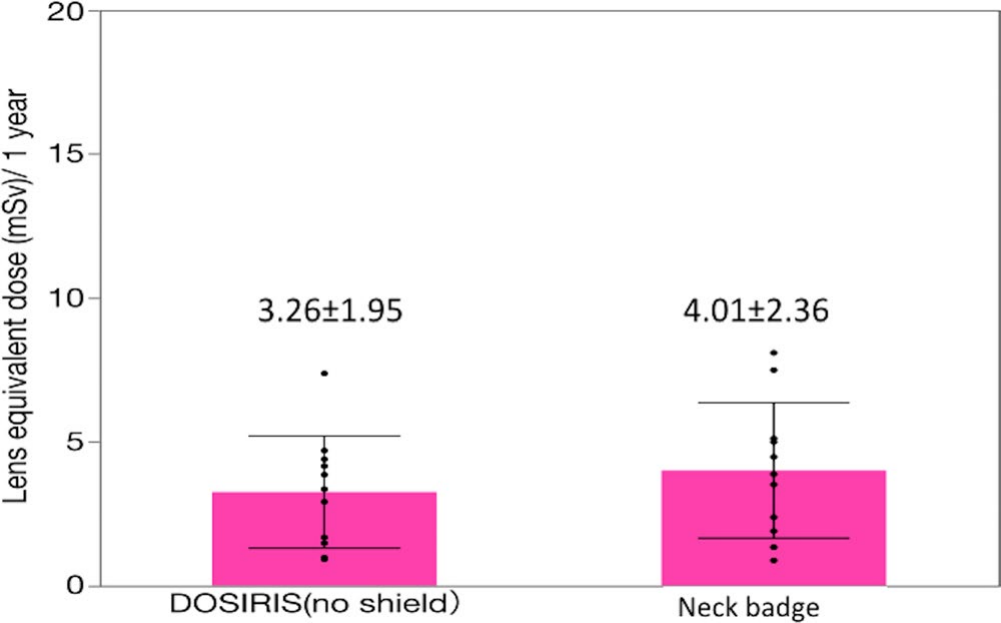
Estimated mean ± SD annual lens dose (EAED) determined by eye dose (DOSIRIS) measurements and neck badge dose measurements in 11 IC nurses. (*P* = 0.39)﻿.

There was a reasonable correlation between the neck badge dose and the eye dosimeter measurements (Fig. 5). Figure 6 shows the EAEDs. No nurse exceeded the equivalent dose limit for the lens of 20 mSv/year. The EAED evaluated using the neck dosimeter (4.0 ± 2.4 mSv/year) was roughly equivalent to the eye dosimeter measurements (3.3 ± 2.0 mSv/year).

## Discussion

The ICRP reviewed epidemiological evidence and suggested that eye tissue reactions such as cataracts occur at radiation doses lower than those considered previously^8, 9^. Many studies have reported the eye dose in IR/IC procedures. It is important to measure the radiation dose to the eye, especially in IR/IC staff. The INTERNATIONAL ATOMIC ENERGY AGENCY (IAEA) recommends that a dosimeter be worn as close as possible to the eye to enable the most accurate monitoring of the eye lens dose^19^.

The ICRP 103 guidelines (2007) recommend that the Hp(3) value should be used to monitor the dose to the lens of the eye^20^. Vanhavere *et al*. and Efstathopoulos *et al*. both measured eye doses encountered during interventional cardiology, but these were the Hp(0.07), not Hp(3), values^21, 22^. Martin *et al*. also measured eye dose exposure during interventional cardiology, but, again, most data were Hp(0.07) values^23^.

Although O’Connor *et al*. reported occupational eye doses during endoscopic retrograde cholangiopancreatography using a direct eye dosimeter, that study involved a small number of subjects (two physicians and two nurses) and covered a 2–month period^13^. In comparison, we present occupational eye dose data from 12 physicians, including those with and without Pb glasses, and 11 nurses over 6 months.

Earlier, the efficacy of lead glasses used to protect the eyes from radiation were evaluated using the Monte Carlo approach^24, 25^. Here, we measured eye doses directly by placing dosimeters above and under the Pb glasses of IC physicians in the clinical setting. The Pb thickness was 0.07 mm; the glasses were lightweight and comfortable. The protective effect was reasonable (ca. 60%). The lightweight glasses were acceptable to the IR/IC physicians, who must often perform long procedures. Glasses of Pb thickness 0.5 mm were heavier, and uncomfortable, especially during long procedures. We thus recommend that IR/IC physicians wear the lightweight glasses.

When using Pb glasses, the annual estimated occupational eye dose measured using the eye dosimeter in the IC physicians was lower than the new maximum allowable radiation limit (20 mSv/year) for radiation workers. However, it was possible for IC physicians who did not wear Pb glasses to exceed the new eye radiation limits. Therefore, IC physicians must wear Pb glasses during procedures.

Koukorava *et al*. reported, in a Monte Carlo study, that ‘wrap-around’ Pb glasses (with 0.07-mm Pb) were the most effective eyewear, reducing the dose by 74%^24^. However, we found that the shielding effect was only 60%, although well-designed glasses (0.07-mm Pb) were used in the clinical setting. Therefore, better-performing Pb glasses may be needed.

As shown in Fig. 2, there was some scatter in the relationship between the neck dose and the eye dosimeter measurements in IC physicians, although the correlation between the neck dose and the eye dosimeter measurements was high (R^2^ = 0.928, 0859). The physician eye dose tended to be overestimated by the neck badge measurements. Furthermore, the eye doses were large in IC physicians. Therefore, for correct evaluation of the lens dose, we recommend measurement of the eye dose in IC physicians using a direct eye dosimeter, such as DOSIRIS^TM^.

In the IC nurses, we found a reasonable correlation (R^2^ = 0.711) between the neck dose and the eye dosimeter measurements, as shown in Fig. 5. For the eye dose evaluations in the nurses, the neck badge measurements were approximately equivalent to the eye dosimeter measurements (Fig. 6). Therefore, the evaluation of lens dose using a neck dosimeter may be reasonable in IC nurses. Nevertheless, the radiation dose in IC nurses was the highest among medical staff except IC physicians^11^. Therefore, we recommend that IC nurses also wear a direct eye dosimeter for correct evaluation of the eye dose.

Additional lead shielding-devices, such as lead acrylic shields suspended from the ceiling, also may provide protection to the physician during IR/IC^26–28^.This paper is on an initial study, so the data were limited, and the accuracy of the statistical analysis may be low.

In summary, we measured the occupational eye dose in IC physicians and nurses in a catheterization laboratory for 6 months. We evaluated glasses with 0.07 mm Pb; these were lightweight and comfortable. We also estimated the eye dose using a neck personal dosimeter. The eye doses among IC staff ranked in the following order: physicians without Pb glasses > physicians with Pb glasses > nurses. The 0.07-mm Pb glasses afforded reasonable protection. In physicians who do not wear Pb glasses, the eye dose may exceed the new regulatory limit for IC staff. Therefore, we recommended that IC physicians wear Pb glasses (0.07-mm Pb lightweight glasses) during procedures. The physician eye dose evaluated using neck dosimeters was overestimated compared with the eye dosimeter measurements. Hence, we recommend that IC physicians use an eye dosimeter such as DOSIRIS^TM^ for correct evaluation of the lens dose. In many IC nurses, evaluation of the eye dose using a neck dosimeter may be reasonable.

Further investigation should examine the relationship between the eye dosimeter dose and dose-related factors, such as dose area product.

## Conclusion

Based on detailed clinical evaluations, we determined the radiation dose in the eyes of IC staff. Furthermore, we evaluated how effectively 0.07-mm Pb lightweight glasses protected the eyes of IC physicians. The new lens dose limit of 20 mSv/year may be exceeded in IR physicians who do not wear Pb glasses. To protect their eyes, IC physicians need to wear Pb glasses. The shielding effect of the glasses (0.07-mm Pb) in a clinical IC setting was approximately 60%. We found good correlations between the neck dosimeter dose and eye dosimeter dose (inside or outside glasses, R^2^ = 0.93 and R^2^ = 0.86, respectively) in physicians. However, the eye dose was overestimated when measured by a neck dosimeter in IC physicians. Correct evaluation of the lens dose (Hp(3)) using an eye dosimeter such as DOSIRIS^TM^ is needed in IC physicians.

The neck and eye dosimeter doses were correlated (R^2^ = 0.71) in nurses. The estimated eye doses in IC nurses were roughly the same between neck and eye dosimeter measurements. Therefore, neck dosimeters may be appropriate for IC nurses. However, use of an eye dosimeter may be required in some IC nurses to measure the lens dose correctly. This study provides useful additional information on eye radiation doses to IR/IC staff, and on the protective effect of lead glasses.
